# Suicide Risk and Living Alone With Depression or Anxiety

**DOI:** 10.1001/jamanetworkopen.2025.1227

**Published:** 2025-03-26

**Authors:** Daa Un Moon, Hyewon Kim, Jin-Hyung Jung, Kyungdo Han, Hong Jin Jeon

**Affiliations:** 1 Department of Psychiatry and Neurosciences, Campus Charité Mitte, Charité Universitätsmedizin Berlin, Berlin, Germany; 2 Psychiatric University Hospital Charité at St. Hedwig Hospital, Berlin, Germany; 3 Department of Psychiatry, Hallym University Sacred Heart Hospital, Anyang, South Korea; 4 Samsung Biomedical Research Institute, Sungkyunkwan University School of Medicine, Suwon, South Korea; 5 Department of Statistics and Actuarial Science, Soongsil University, Seoul, South Korea; 6 Depression Center, Department of Psychiatry, Samsung Medical Center, Sungkyunkwan University School of Medicine, Seoul, South Korea; 7 Department of Health Sciences & Technology, Samsung Advanced Institute for Health Sciences & Technology, Sungkyunkwan University, Seoul, South Korea; 8 Department of Medical Device Management & Research, Samsung Advanced Institute for Health Sciences & Technology, Sungkyunkwan University, Seoul, South Korea; 9 Department of Clinical Research Design & Evaluation, Samsung Advanced Institute for Health Sciences & Technology, Sungkyunkwan University, Seoul, South Korea

## Abstract

**Importance:**

Living alone and mental health disorders, including depression and anxiety, are associated with high suicide risk, but their combined impact remains underexplored.

**Objective:**

To examine the association of living arrangements and depression or anxiety with suicide risk.

**Design, Setting, and Participants:**

This population-based cohort study used data from the Korean National Health Insurance Service database from January 1, 2009, to December 31, 2021, that included adults aged 20 years or older who participated in the General Health Screening Program in Korea in 2009. Individuals with incomplete data and those who died by suicide within the first year were excluded to minimize the possibility of reverse causation. Data were analyzed from December 28, 2023, to December 27, 2024.

**Exposures:**

Living arrangements, categorized as living alone (≥5 years) or living with others, and depression and anxiety, determined using *International Statistical Classification of Diseases and Related Health Problems, Tenth Revision* codes.

**Main Outcomes and Measures:**

The primary outcome was death by suicide, identified through national death records. Multivariable Cox proportional hazards regression models were used to estimate adjusted hazard ratios (AHRs) and 95% CIs for suicide risk.

**Results:**

The study included 3 764 279 adults (mean [SD] age, 47.2 [14.0] years; 55.8% male). Of these individuals, 112 460 (3.0%) had depression, 232 305 (6.2%) had anxiety, and 319 993 (8.5%) lived alone. Compared with individuals who had neither depression nor anxiety nor lived alone, individuals living alone with both depression and anxiety exhibited a 558% increased risk of suicide (AHR, 6.58 [95% CI, 4.86-8.92]). Living alone with depression was associated with a 290% increased risk (AHR, 3.91 [95% CI, 2.96-5.16]), and living alone with anxiety was associated with a 90% increased risk (AHR, 1.90 [95% CI, 1.48-2.43]). The AHRs were higher for the association between living alone and suicide among middle-aged individuals (aged 40 to 64 years) and men compared with other demographic groups.

**Conclusions and Relevance:**

In this cohort study of 3 764 279 individuals, living alone with depression or anxiety was associated with an increased risk of suicide, particularly among middle-aged individuals and men. These findings underscore the importance of preventing mental illness, such as depression and anxiety, while addressing living arrangements as a critical factor in suicide risk assessments for individuals with these conditions.

## Key Points

**Question:**

What is the association between living arrangements, depression or anxiety, and suicide risk?

**Findings:**

In this cohort study of 3 764 279 adults, living alone with depression or anxiety was associated with a significantly higher risk of suicide, particularly among middle-aged individuals (aged 40 to 64 years) and men.

**Meaning:**

The findings of this study suggest that living alone, when combined with depression or anxiety, was associated with an increased risk of suicide, highlighting the importance of targeted mental health interventions and suicide-prevention strategies.

## Teaser

This cohort study examines the association of suicide risk with living arrangements and depression or anxiety among adults in Korea.

## Introduction

Suicide is a critical global health issue that is responsible for more than 700 000 deaths annually.^1^ While suicide rates vary across regions, South Korea (hereinafter referred to as Korea) consistently reported the highest suicide rate among Organisation for Economic Co-operation and Development countries from 2003 to 2023, with 24.1 suicides per 100 000 individuals.^2^ This trend may be attributed to rapid industrialization and urbanization, disrupting traditional social support systems,^3^ along with academic and workplace pressures, mental health stigma, and job insecurity.^4,5,6,7,8^

Suicide arises from complex associations among biological, psychological, socioeconomic, and clinical factors,^9,10^ with mental disorders such as depression and anxiety being well-established risk factors.^11,12,13^ Living arrangements, particularly living alone, have emerged as critical social determinants of mental and physical health.^14^ Although living alone is a physical state, it is often conflated with social isolation and loneliness, which are distinct yet interconnected concepts.^15,16^ Living alone refers to residing by oneself physically but does not inherently indicate social isolation (eg, a lack of social connections and interactions).^17^ However, living alone can often lead to social isolation, which has been associated with a range of adverse health outcomes, including psychiatric disorders, dementia, poor nutrition, diabetes, and cardiovascular disease.^18,19,20,21,22^ Social isolation can intensify feelings of loneliness—a subjective emotional state characterized by a sense of being alone and disconnected—and may exacerbate feelings of hopelessness and key psychological precursors to suicidal behavior.^9,23^ Studies have indicated that living alone significantly raises the risk of all-cause mortality and is comparable with the increased risks associated with social isolation and loneliness.^17^

Living alone has also been associated with suicide risk across various populations, including those with mental disorders or prior suicide attempts.^23,24,25,26,27,28,29^ In recent decades, living arrangements have undergone substantial changes, with a notable increase in 1-person households across high-income countries, accounting for more than one-fourth of all households.^14,30^ Similarly, in Korea, the proportion of 1-person households reached 34.5% of all households in 2022 (approximately 7.5 million people),^31^ reflecting broader trends of urbanization, aging populations, and shifting family structures, including a decline in multigenerational households.^32,33^ Weakening traditional family support systems and rising divorce rates further highlight societal changes in Korea.^34^

While prior research has linked psychiatric disorders and suicide and explored the health implications of living alone, gaps remain in understanding their combined impact. Studies on living alone often neglect mental health,^17,35^ while research on depression or anxiety rarely considers living environments,^13^ overlooking a possible combined effect on suicide risk. This study aimed to address this gap by examining the associations among living arrangements, mental health conditions (depression and anxiety), and suicide risk in a large national cohort to inform targeted suicide prevention strategies.

## Methods

### Data Source and Study Population

This cohort study used data from the Korean National Health Insurance Service (NHIS), which covers 97% of Korea’s population through a mandatory health insurance program, from January 1, 2009, to December 31, 2021. The Medical Aid Program covers the remaining 3% of the population with the lowest income. The NHIS conducts biennial health screenings for insured individuals, which include laboratory tests and self-reported health questionnaires. The NHIS database, established in 2002, contains demographic details, health examination results, disease diagnoses, and medical treatment records based on the *International Statistical Classification of Diseases, Tenth Revision, Clinical Modification*. Since 2015, researchers have been granted access to the database upon the official review committee’s approval of their study protocols. This study was conducted according to the Declaration of Helsinki^36^ and was approved by the Institutional Review Board of Soongsil University. Owing to the use of deidentified data, the requirement for informed consent was waived. We followed the Strengthening the Reporting of Observational Studies in Epidemiology (STROBE) reporting guideline.

**Figure 1.**
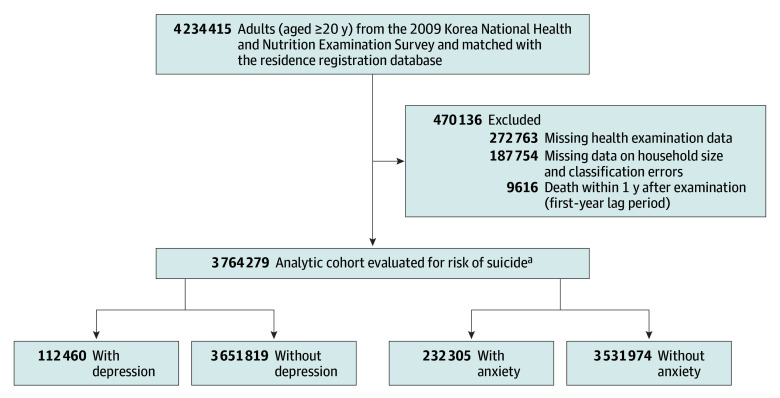
Flowchart of Study Population Selection ^a^Depression (yes or no) and anxiety (yes or no) classifications are independent. Each category sums to the total cohort size of individuals (N = 3 764 279).

The initial cohort comprised adults aged 20 years or older who participated in the General Health Screening Program in Korea in 2009 (Figure 1). From this initial population, exclusions were made among individuals with incomplete health examination data. Further exclusions were made for individuals with absent data on household size or with discrepancies in their household size classification; this included those erroneously recorded as single household members and listed as dependents. A 1-year lag period was instituted to minimize possible reverse causality,^37^ leading to the exclusion of individuals who died by suicide within the first year of observation. After applying these exclusion criteria, the final cohort was established.

### Exposure

Living arrangements were defined based on the registration information from the NHIS. Individuals were considered living alone if registered as a 1-person household for 5 or more years at baseline to minimize misclassification of transient living arrangements.^37^ Living arrangements were dichotomized into 1 adults living alone (living alone) or adults living with others, which may include both family members and nonfamily individuals (living together). Depression and anxiety were identified using NHIS claims within the year preceding the health examination to capture more recent and likely active psychiatric conditions based on health care utilization.

### Outcome

The primary outcome of the study was death by suicide, identified through death records obtained from Statistics Korea, the National Statistical Office. Suicide cases were determined using the *International Statistical Classification of Diseases and Related Health Problems, Tenth Revision* (*ICD-10*) codes X60 to X84, as listed in the cause of death. Deaths from nonsuicide causes were censored to account for competing risks. The population was followed from baseline to the date of suicide, censoring data (eg, outmigration), or until December 31, 2021.

### Covariates

The study considered a range of demographic, socioeconomic, lifestyle, and clinical factors (eTable 1 in Supplement 1). Demographic data were obtained from individual NHIS records. Economic status was assessed using health insurance premiums as an indicator of income in Korea; individuals on medical aid or in the lowest income quartile were categorized as having a low-income status. Information about lifestyle, such as smoking habits, alcohol consumption, and physical activity, was gathered from standardized health-screening questionnaires. Clinical factors were determined based on baseline comorbidities identified through *ICD-10* codes, pharmacy records, and physical examination results supported by medical service claims. Laboratory measures were obtained after an overnight fast exceeding 8 hours.

### Statistical Analysis

Data were analyzed from December 28, 2023, to December 27, 2024. Baseline characteristics of the study population were summarized using means with SDs for continuous variables and frequencies and percentages for categorical variables. We categorized the population into 8 groups based on depression (yes or no), anxiety (yes or no), and living arrangement (living alone or living together). The comparison of continuous variables between different exposure groups was conducted using analysis of variance, and the χ^2^ test was applied to compare categorical variables. The incidence rates of suicide were expressed as events per 1000 person-years of follow-up. The baseline time for the analysis was set as the day of the health checkup conducted in 2009.

Survival analysis was performed using Kaplan-Meier curves to illustrate the cumulative event rates of suicide, with the log-rank test comparing these rates. We used multivariable Cox proportional hazards regression models to assess the association of living arrangements and psychiatric disorders (depression, anxiety) with suicide risk, used individuals living together with no depression or anxiety as the reference group, and provided adjusted hazard ratios (AHRs) with 95% CIs. The proportional hazards assumption was satisfied for all variables. The analysis included 6 models with increasing adjustments: model 1 was unadjusted; model 2 adjusted for sex and age; model 3 added economic and lifestyle factors (income, smoking status, alcohol consumption, and physical activity) and clinical characteristics (body mass index, diabetes, hypertension, dyslipidemia, and chronic kidney disease); and model 4 added cancer. These chronic illnesses were included due to their association with increased psychological stress, social isolation, reduced quality of life, and suicide risk.^38,39,40^ Model 5 was further adjusted for psychiatric disorders (posttraumatic stress disorder, schizophrenia spectrum disorder, substance use disorder, and bipolar disorder), and model 6 included obsessive-compulsive and personality disorders. Interaction terms tested whether the association of living alone with suicide risk differed by the presence of depression or anxiety. Sensitivity analyses were stratified by follow-up duration (1 to <5 years and 5 to <9 years), duration of living alone (1 year, 2-4 years, and ≥5 years), and exclusion of individuals with missing data. Subgroup analyses by sex and age explored demographic variations in risk.

Statistical analyses were carried out using SAS, version 9.4 (SAS Institute Inc). A 2-sided *P* value <.05 was considered indicative of statistical significance.

## Results

### Baseline Characteristics of the Study Population

The initial population comprised 4 234 415 individuals, of whom 470 136 were excluded (272 763 with incomplete health examination data, 187 754 with discrepancies in household size and classification, and 9619 who died by suicide within the 1-year lag period). The final study cohort included 3 764 279 adults (mean [SD] age, 47.2 [14.0] years; 44.2% female and 55.8% male). Of these, 112 460 (3.0%) had depression, 232 305 (6.2%) had anxiety, 3 444 286 (91.5%) lived together, and 319 993 (8.5%) lived alone (Table 1). Among those living together, 104 902 (3.0%) were diagnosed with depression compared with 7558 (2.4%) living alone. In the case of anxiety, 216 432 (6.3%) individuals living together were diagnosed compared with 15 873 (5.0%) living alone. Individuals with depression or anxiety, irrespective of living arrangements, were generally older, female, and from low-income brackets. Furthermore, these individuals exhibited higher prevalence rates of comorbid conditions such as diabetes, hypertension, dyslipidemia, and cardiovascular disease compared with those without these mental health disorders.

**Table 1.** Baseline Characteristics of the Study Population^a^

Characteristic	Total cohort (N = 3 764 279)	Living together		Living alone		*P* value	Living together		Living alone		*P* value
		Without depression (n = 3 339 384)	With depression (n = 104 902)	Without depression (n = 312 435)	With depression (n = 7558)		Without anxiety (n = 3 227 854)	With anxiety (n = 216 432)	Without anxiety (n = 304 120)	With anxiety (n = 15 873)	
Sex											
Female	1 663 757 (44.2)	1 431 349 (42.9)	66 629 (63.5)	160 589 (51.4)	5190 (68.7)	<.001	1 362 656 (42.2)	135 322 (62.5)	154 810 (50.9)	10 969 (69.1)	<.001
Male	2 100 522 (55.8)	1 908 035 (57.1)	38 273 (36.5)	151 846 (48.6)	2368 (31.3)		1 865 198 (57.8)	81 110 (37.5)	149 310 (49.1)	4904 (30.9)	
Age, mean (SD), y	47.2 (14.0)	47.3 (14.0)	57.1 (13.1)	42.5 (13.0)	52.8 (14.4)	<.001	47.0 (13.9)	56.2 (13.3)	42.3 (12.9)	52.4 (14.5)	<.001
Age group, y											
20-39	1 180 596 (31.4)	1 016 920 (30.5)	9345 (8.9)	152 841 (48.9)	1490 (19.7)	<.001	1 003 831 (31.1)	22 434 (10.4)	151 061 (49.7)	3270 (20.6)	<.001
40-64	2 092 622 (55.6)	1 891 354 (56.6)	62 215 (59.3)	134 844 (43.2)	4209 (55.7)		1 824 084 (56.5)	129 485 (59.8)	130 292 (42.8)	8761 (55.2)	
≥65	491 061 (13.1)	431 110 (12.9)	33 342 (31.8)	24 750 (7.92)	1859 (24.6)		399 939 (12.4)	64 513 (29.8)	22 767 (7.5)	3842 (24.2)	
Low income^b^	730 165 (19.4)	616 435 (18.5)	18 926 (18.0)	91 501 (29.3)	3303 (43.7)	<.001	595 025 (18.4)	40 336 (18.6)	88 053 (29.0)	6751 (42.5)	<.001
Smoking status											
None	2 216 940 (58.9)	1 943 042 (58.2)	76 512 (72.9)	191 960 (61.4)	5426 (71.8)	<.001	1 861 489 (57.7)	158 065 (73.0)	185 765 (61.1)	11 621 (73.2)	<.001
Former	551 599 (14.7)	505 253 (15.1)	13 074 (12.5)	32 481 (10.4)	791 (10.5)		491 182 (15.2)	27 145 (12.5)	31 714 (10.4)	1558 (9.8)	
Current	995 740 (26.5)	891 089 (26.7)	15 316 (14.6)	87 994 (28.2)	1341 (17.7)		875 183 (27.1)	31 222 (14.4)	86 641 (28.5)	2694 (17.0)	
Alcohol consumption^c^	1 839 357 (48.9)	1 651 562 (49.5)	29 358 (28.0)	156 044 (49.9)	2393 (31.7)	<.001	1 615 637 (50.1)	65 283 (30.2)	153 258 (50.4)	5179 (32.6)	<.001
Regular physical activity^d^	676 613 (18.0)	606 866 (18.2)	19 678 (18.8)	48 757 (15.6)	1312 (17.4)	<.001	585 679 (18.1)	40 865 (18.9)	47 461 (15.6)	2608 (16.4)	<.001
Comorbidities											
Obesity^e^	1 230 330 (32.7)	1 104 786 (33.1)	36 366 (34.7)	86 861 (27.8)	2317 (30.7)	<.001	1 066 478 (33.0)	74 674 (34.5)	84 359 (27.7)	4819 (30.4)	<.001
Diabetes	326 895 (8.7)	290 174 (8.7)	15 698 (15.0)	20 071 (6.4)	952 (12.6)	<.001	277 574 (8.6)	28 298 (13.1)	19 210 (6.3)	1813 (11.4)	<.001
Hypertension	957 084 (25.4)	851 010 (25.5)	43 418 (41.4)	60 086 (19.2)	2570 (34.0)	<.001	806 172 (25.0)	88 256 (40.8)	57 114 (18.8)	5542 (34.9)	<.001
Dyslipidemia	649 965 (17.3)	570 864 (17.1)	31 651 (30.2)	45 418 (14.5)	2032 (26.9)	<.001	541 934 (16.8)	60 581 (28.0)	43 404 (14.3)	4046 (25.5)	<.001
Chronic kidney disease	262 158 (7.0)	230 935 (6.9)	12 496 (11.9)	18 010 (5.8)	717 (9.5)	<.001	219 735 (6.8)	23 696 (11.0)	17 174 (5.7)	1553 (9.8)	<.001
Cardiovascular disease	70 708 (1.9)	57 293 (1.7)	9754 (9.3)	3133 (1.0)	528 (7.0)	<.001	51 813 (1.6)	15 234 (7.0)	2849 (0.9)	812 (5.1)	<.001
Myocardial infarction	14 435 (0.4)	12 195 (0.4)	1419 (1.4)	740 (0.2)	81 (1.1)	<.001	10 885 (0.3)	2729 (1.3)	662 (0.2)	159 (1.0)	<.001
Stroke	57 789 (1.5)	46 244 (1.4)	8639 (8.2)	2447 (0.8)	459 (6.1)	<.001	41 956 (1.3)	12 927 (6.0)	2237 (0.7)	669 (4.2)	<.001
Cancer	47 635 (1.3)	42 163 (1.3)	2783 (2.7)	2534 (0.8)	155 (2.1)	<.001	39 937 (1.2)	5009 (2.3)	2414 (0.8)	275 (1.7)	<.001
Anxiety	232 305 (6.2)	180 341 (5.4)	36 091 (34.4)	13 402 (4.3)	2471 (32.7)	<.001	0	216 432 (100.0)	0	15 873 (100.0)	<.001
Depression	112 460 (3.0)	0	104 902 (100.0)	0	7558 (100.0)	<.001	68 811 (2.1)	36 091 (16.7)	5087 (1.7)	2471 (15.6)	<.001
Anthropometric measures, mean (SD)											
Height, cm	164.0 (9.2)	164.2 (9.2)	159.4 (8.9)	163.9 (8.9)	159.6 (9.0)	<.001	164.3 (9.2)	159.7 (9.0)	164.0 (8.9)	159.6 (9.0)	<.001
Weight, cm	64.0 (11.7)	64.3 (11.6)	60.8 (10.5)	62.7 (12.0)	60.0 (10.7)	<.001	64.4 (11.7)	61.0 (10.5)	62.8 (12.0)	60.0 (10.8)	<.001
BMI	23.7 (3.2)	23.8 (3.2)	23.9 (3.2)	23.2 (3.3)	23.5 (3.3)	<.001	23.7 (3.2)	23.9 (3.2)	23.2 (3.3)	23.5 (3.3)	<.001
Waist circumference, cm	80.3 (9.1)	80.5 (9.1)	81.1 (8.9)	78.3 (9.5)	79.3 (9.3)	<.001	80.5 (9.1)	81.0 (8.8)	78.3 (9.5)	79.2 (9.3)	<.001
Systolic blood pressure, mm Hg	122.5 (15.0)	122.7 (15.0)	123.8 (15.6)	120.6 (15.0)	121.9 (15.7)	<.001	122.6 (15.0)	124.1 (15.5)	120.5 (15.0)	122.4 (15.8)	<.001
Diastolic blood pressure, mm Hg	76.4 (10.1)	76.5 (10.1)	76.5 (10.0)	75.5 (10.2)	75.6 (10.0)	<.001	76.4 (10.1)	76.7 (10.0)	75.5 (10.1)	75.9 (10.2)	<.001
Laboratory values											
Glucose, mg/dL, mean (SD)	97.3 (23.8)	97.4 (23.8)	100.3 (26.2)	95.2 (23.1)	98.9 (27.4)	<.001	97.4 (23.8)	99.6 (24.8)	95.1 (23.1)	98.2 (24.9)	<.001
Total cholesterol, mg/dL, mean (SD)	195.0 (36.8)	195.0 (36.7)	198.2 (39.2)	193.6 (36.5)	198.2 (39.5)	<.001	194.9 (36.6)	198.0 (38.5)	193.5 (36.5)	197.6 (38.6)	<.001
High-density lipoprotein cholesterol, mg/dL, mean (SD)	56.0 (27.7)	55.9 (27.8)	56.2 (33.3)	57.3 (24.9)	56.9 (26.8)	<.001	55.9 (27.7)	56.2 (32.2)	57.3 (24.6)	57.4 (29.8)	<.001
Low-density lipoprotein cholesterol, mg/dL, mean (SD)	113.5 (38.6)	113.5 (38.6)	116.4 (40.5)	111.6 (37.6)	115.1 (37.4)	<.001	113.5 (38.6)	116.4 (39.4)	111.5 (37.6)	115.2 (38.0)	<.001
Glomerular filtration rate, mL/min/1.73 m^2^, mean (SD)	87.6 (45.9)	87.5 (45.8)	83.2 (36.4)	89.6 (50.2)	85.1 (39.0)	<.001	87.7 (46.1)	83.8 (36.6)	89.8 (50.6)	85.0 (36.1)	<.001
Triglycerides, mg/dL, geometric mean (95% CI)	112.9 (112.8-113.0)	113.3 (113.2-113.4)	118.9 (118.5-119.3)	106.9 (106.7-107.1)	114.3 (112.9-115.8)	<.001	113.2 (113.2-113.3)	116.8 (116.6-117.1)	106.8 (106.6-107.0)	112.0 (111.0-113.0)	<.001

Abbreviation: BMI, body mass index (calculated as weight in kilograms divided by height in meters squared).

SI conversion factors: to convert glucose to millimoles per liter, multiply by 0.0555; total cholesterol, high-density lipoprotein cholesterol, and low-density lipoprotein cholesterol to millimoles per liter, multiply by 0.0259; and triglycerides to millimoles per liter, multiply by 0.0113.

^a^ Data are presented as No. (%) of individuals unless otherwise indicated.

^b^ Individuals in the bottom 25% income bracket based on health insurance premiums in South Korea.

^c^ Individuals reporting any frequency of alcohol consumption.

^d^ Individuals engaging in moderate-intensity exercise for at least 30 minutes, 5 or more days per week or vigorous activity for at least 20 minutes, 3 or more days per week.

^e^ Defined as a BMI of 25 or higher.

### Mortality and Suicide Rates

eTable 2 in Supplement 1 outlines data on total mortality, suicide rates, and follow-up duration, categorized by living arrangements and psychiatric conditions. Among the total cohort, there were 235 458 deaths (6.3%). Suicide accounted for 11 648 (0.3%) of all deaths, with a higher incidence among individuals living alone with depression (1.2%) and anxiety (0.7%) compared with those living with others and without these psychiatric conditions. The mean (SD) follow-up period for the entire cohort was 11.05 (1.46) years.

### Survival Analysis

**Figure 2.**
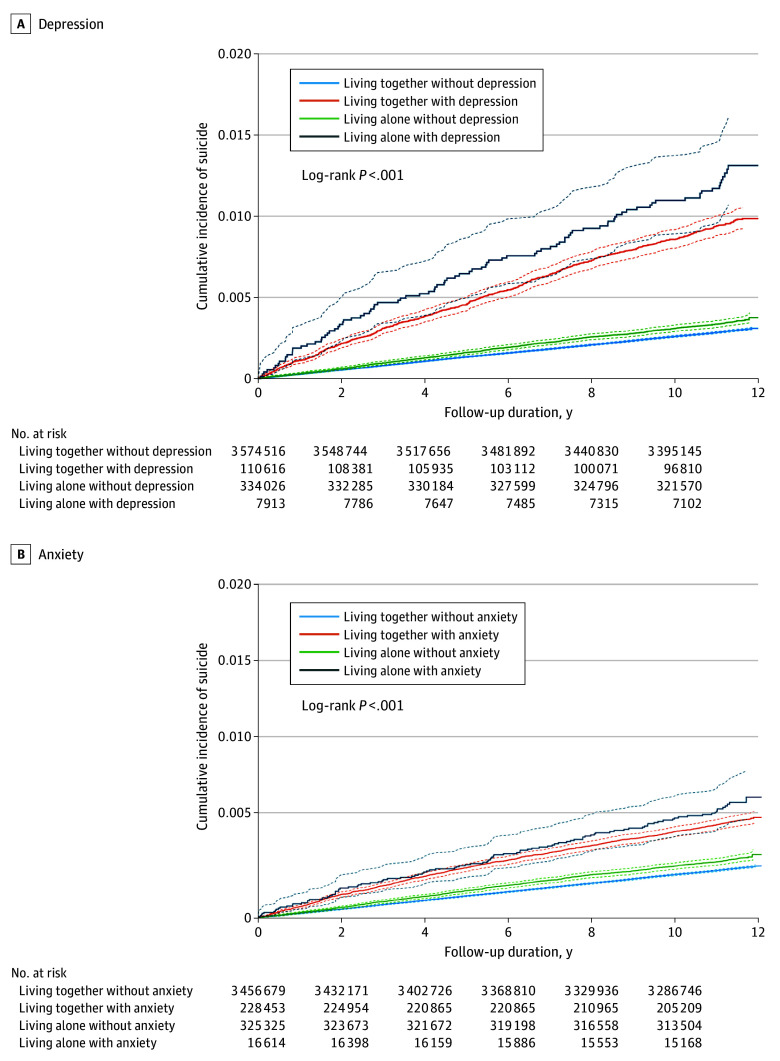
Cumulative Incidence of Suicide Stratified by Living Arrangement and Psychiatric Condition Kaplan-Meier estimates for the cumulative incidence of suicide over 11 years. Individuals who died by suicide within the first year after baseline were excluded from the analysis; the Kaplan-Meier curves, however, are plotted from time 0 for visualization purposes. Dotted lines indicate 95% CIs.

Figure 2 displays Kaplan-Meier survival curves for the cumulative incidence of suicide, stratified by living arrangement and the presence of depression or anxiety. Individuals living alone with depression had the highest cumulative incidence of suicide, reaching 1.2% by the end of the follow-up period, followed by those living together with depression. Similarly, for anxiety, individuals living alone showed the highest incidence, reaching 0.7%, followed by those living together with anxiety.

### Cox Proportional Hazards Regression

The results of the multivariable Cox proportional hazards regression models are presented in Table 2. Individuals with both depression and anxiety living alone exhibited the highest risk of suicide, with a 558% increased risk (AHR, 6.58 [95% CI, 4.86-8.92]) compared with the reference group (no depression, no anxiety, and living together), even after adjustments for multiple covariates, including demographic, lifestyle, and clinical factors (model 4). For individuals with depression but no anxiety, those living alone had a 290% increased risk (AHR, 3.91 [95% CI, 2.96-5.16]), while those living together had a 198% increased risk (AHR, 2.98 [95% CI, 2.74-3.25]). For individuals with anxiety but no depression, those living alone had a 90% increased risk of suicide (AHR, 1.90 [95% CI, 1.48-2.43]) compared with the reference group, while those living together had a 64% increased risk (AHR, 1.64 [95% CI, 1.52-1.76]). Individuals living alone without depression or anxiety had a 44% increased risk (AHR, 1.44 [95% CI, 1.35-1.54]). The association of living arrangements combined with depression or anxiety with suicide risk was significant across all models, persisting after adjusting for psychiatric comorbidities (models 5 and 6).

**Table 2.** Suicide Risk by Living Arrangement and Psychiatric Condition

Living arrangement and psychiatric condition	Individuals, No.	Events, No.	Follow-up duration, person-years	Incidence rate^a^	Model, AHR (95% CI)					
					1^b^	2^c^	3^d^	4^e^	5^f^	6^g^
**Living together**										
Without depression or anxiety	3 159 043	8716	34 982 431	0.25	1 [Reference]	1 [Reference]	1 [Reference]	1 [Reference]	1 [Reference]	1 [Reference]
Without depression but with anxiety	180 341	820	1 956 092	0.42	1.68 (1.57-1.81)	1.62 (1.51-1.74)	1.64 (1.52-1.76)	1.64 (1.52-1.76)	1.60 (1.48-1.72)	1.59 (1.48-1.71)
With depression but without anxiety	68 811	573	736 187	0.78	3.12 (2.87-3.40)	2.97 (2.73-3.23)	2.99 (2.74-3.25)	2.98 (2.74-3.25)	2.63 (2.41-2.87)	2.61 (2.39-2.85)
With depression and anxiety	36 091	382	381 676	1.00	4.012 (3.62-4.45)	3.77 (3.40-4.19)	3.85 (3.47-4.27)	3.83 (3.45-4.25)	3.26 (2.93-3.62)	3.20 (2.88-3.57)
**Living alone**										
Without depression or anxiety	299 033	1002	3 327 562	0.30	1.21 (1.13-1.29)	1.56 (1.46-1.66)	1.44 (1.35-1.54)	1.44 (1.351-1.542)	1.44 (1.35-1.54)	1.44 (1.35-1.54)
Without depression but with anxiety	13 402	63	146 242	0.43	1.728 (1.35-2.21)	2.05 (1.60-2.63)	1.90 (1.48-2.43)	1.90 (1.48- 2.43)	1.84 (1.44-2.36)	1.84 (1.43-2.35)
With depression but without anxiety	5087	50	55 052	0.91	3.64 (2.76-4.81)	4.28 (3.24-5.65)	3.91 (2.96-5.16)	3.91 (2.96- 5.16)	3.38 (2.55-4.46)	3.33 (2.52-4.41)
With depression and anxiety	2471	42	26 336	1.60	6.40 (4.72-8.66)	7.22 (5.33-9.79)	6.60 (4.87-8.94)	6.58 (4.86- 8.92)	5.68 (4.20-7.71)	5.63 (4.15-7.64)

Abbreviation: AHR, adjusted hazard ratio.

^a^ Incidence per 1000 person-years.

^b^ Not adjusted; *P* < .001.

^c^ Adjusted for sex and age; *P* < .001.

^d^ Adjusted for sex, age, income, smoking status, alcohol consumption, regular physical activity, body mass index (BMI), diabetes, hypertension, dyslipidemia, and chronic kidney disease; *P* < .001.

^e^ Adjusted for sex, age, income, smoking status, alcohol consumption, regular physical activity, BMI, diabetes, hypertension, dyslipidemia, chronic kidney disease, and cancer; *P* < .001.

^f^ Adjusted for sex, age, income, smoking status, alcohol consumption, regular physical activity, BMI, diabetes, hypertension, dyslipidemia, chronic kidney disease, cancer, posttraumatic stress disorder, schizophrenia spectrum disorder, substance use disorder, and bipolar disorder; *P* < .001.

^g^ Adjusted for sex, age, income, smoking status, alcohol consumption, regular physical activity, BMI, diabetes, hypertension, dyslipidemia, chronic kidney disease, cancer, posttraumatic stress disorder, schizophrenia spectrum disorder, substance use disorder, bipolar disorder, obsessive-compulsive disorder, and personality disorder; *P* < .001.

Interaction terms between living arrangements and depression or anxiety were not significant, indicating no association with suicide risk (eTable 3 in Supplement 1). Further analyses supported the consistency of findings. Stratification by follow-up duration (1 to <5 years and 5 to <9 years) showed that the AHRs were highest for the associations during the first follow-up period, with slight attenuation over time but consistent patterns overall (eTable 4 in Supplement 1). Sensitivity analyses by duration of living alone (1 year, 2-4 years, and ≥5 years) and exclusion of individuals with missing data yielded similar associations (eTables 5 and 6 in Supplement 1).

### Subgroup Analysis

The subgroup analysis showed variations in suicide risk based on sex and age (Table 3), with significant associations observed for both. For individuals with depression, those living alone had the highest risk, especially among males (AHR, 4.32 [95% CI, 3.30-5.67]; *P* < .001 for interaction) and those aged 40 to 64 years (AHR, 6.02 [95% CI, 4.70-7.71]; *P* < .001 for interaction). In contrast, individuals with depression living together had lower risk, with females (AHR, 2.99 [95% CI, 2.68-3.34]) and those aged 20 to 39 years (AHR, 3.66 [95% CI, 2.87-4.66]) showing the highest risks in their respective groups. Similarly, for anxiety, the highest risk was observed in individuals living alone, particularly males (AHR, 2.07 [95% CI, 1.59-2.70]) and those aged 40 to 64 years (AHR, 2.61 [95% CI, 2.03-3.35]). Those living together with anxiety had lower risks, with females (AHR, 1.56 [95% CI, 1.40-1.72]) and the 20- to 39-year age group (AHR, 1.57 [95% CI, 1.24-1.97]) having the highest risk in their respective groups.

**Table 3.** Suicide Risk by Psychiatric Condition and Living Arrangement in Subgroups by Sex and Age^a^

Psychiatric condition and living arrangement	Sex, AHR (95% CI)		Age, AHR (95% CI), y		
	Female	Male	20-39	40-64	≥65
**Depression**					
Living together without depression	1 [Reference]	1 [Reference]	1 [Reference]	1 [Reference]	1 [Reference]
Living together with depression	2.99 (2.68-3.34)	2.59 (2.37-2.83)	3.66 (2.87-4.66)	3.16 (2.87-3.47)	2.19 (1.96-2.44)
Living alone without depression	1.16 (1.01-1.32)	1.52 (1.42-1.64)	1.24 (1.11-1.39)	1.68 (1.54-1.84)	1.06 (0.89-1.26)
Living alone with depression	3.71 (2.70-5.10)	4.32 (3.30-5.67)	4.00 (2.21-7.24)	6.02 (4.70-7.71)	1.75 (1.09-2.83)
**Anxiety**					
Living together without anxiety	1 [Reference]	1 [Reference]	1 [Reference]	1 [Reference]	1 [Reference]
Living together with anxiety	1.56 (1.40-1.72)	1.52 (1.41-1.65)	1.57 (1.24-1.97)	1.53 (1.40-1.68)	1.51 (1.38-1.66)
Living alone without anxiety	1.14 (1.00-1.31)	1.54 (1.44-1.66)	1.24 (1.10-1.38)	1.70 (1.56-1.86)	1.05 (0.88-1.25)
Living alone with anxiety	2.00 (1.51-2.66)	2.07 (1.59-2.70)	1.87 (1.06-3.30)	2.61 (2.03-3.35)	1.41 (0.98-2.02)

Abbreviation: AHR, adjusted hazard ratio.

^a^ Values were calculated after adjusting for age, sex, income, smoking status, alcohol consumption, regular physical activity, body mass index, diabetes, hypertension, dyslipidemia, chronic kidney disease, cancer, depression, and anxiety. *P* < .001 for interaction for sex and age.

## Discussion

In this national cohort study of 3 764 279 individuals, we examined the association between living arrangements, depression, anxiety, and suicide risk. Our study yielded 3 primary findings: (1) individuals with depression or anxiety living alone were associated with an increased risk of suicide, (2) the highest risk was observed in individuals living alone with both depression and anxiety, and (3) males and individuals aged 40 to 64 years living alone with depression or anxiety faced the highest suicide risk. These findings remained consistent after adjustments for demographic, lifestyle, and clinical factors, as well as across different follow-up periods, highlighting the combined association of living arrangements and mental health conditions with suicide risk.

Previous research has associated living alone and social isolation with the development of depression and anxiety.^16,21,22,41^ Studies have also demonstrated an association between living alone and an increased risk of suicide.^9,23,28,29,42^ Additionally, the well-established association between depression and/or anxiety with suicide highlights the significant effects of these mental health conditions associated with suicidal behavior.^10,12,13^ Research further indicates that social isolation and mental health conditions are jointly associated with the vulnerability of suicide behavior^26,27,43,44^ and mortality.^17^ Consistent with these findings, our study showed that AHRs were higher for the association between depression or anxiety and suicide risk than were those for living alone. However, their combined, additive association significantly increased suicide risk, emphasizing the importance of addressing these factors together in prevention strategies.

Psychosocially, individuals living alone may lack social support, which is critical for mitigating the effects of depression and anxiety.^22,23^ The absence of social interaction due to living alone could intensify feelings of loneliness and hopelessness, which are key psychological precursors to suicide.^9,10,23^ In Korea, sociocultural factors such as mental health stigma may discourage help-seeking behaviors, exacerbating isolation.^4,6^ Additionally, academic and workplace pressures, social expectations of success, and job insecurity coupled with cultural norms emphasizing family honor could further contribute to psychological distress among individuals living alone.^7,8^ Biologically, chronic stress from living alone and social isolation may dysregulate the hypothalamic-pituitary-adrenal axis and may induce systemic inflammation, which are both associated with depression, anxiety, and suicide risk. Stress-related epigenetic changes and neurotransmitter dysregulation may further exacerbate suicide risk.^10^

The findings of our subgroup analyses indicate that increased suicide risk is more pronounced among middle-aged individuals (aged 40 to 64 years) and men, aligning with results from previous research.^42^ A recent meta-analysis found that living alone increased mortality to a greater extent in males than in females.^45^ Similarly, a study on Korean adults older than 65 years reported an association between living alone and suicidal ideation in men but not in women.^46^ Studies from the UK and Germany also noted that living alone raised the risk of suicide and mortality in men.^47,48^ This sex disparity may be due to men’s higher overall suicide mortality rates, which are approximately 2.3 times those of women in Korea.^1,49^ Men’s preference for more lethal methods and barriers to seeking help may exacerbate the risks of living alone.^50^ Regarding age, living alone has been associated with increased mortality and suicide risk, particularly in middle-aged individuals.^45,51^ This association may stem from an increased risk of psychiatric morbidity associated with living alone in midlife.^22,52^ Motivations and circumstances for living alone vary across life stages. In midlife, living alone often results from separation, divorce, or widowhood, which may amplify social isolation and psychological distress.^53^ This period is also marked by shifts in family structures, career transitions, and changes in health status, which could further strain social relationships.^54^ In contrast, younger adults may perceive living alone as a sign of independence, whereas older adults typically receive social support from children or caregivers, mitigating associated risks.^55^ Additionally, older adults may prioritize a few high-quality relationships, while younger adults may find satisfaction in broader social networks.^56^

The findings of this study underscored the importance of considering both living arrangements and mental health conditions in suicide-prevention strategies. Individuals living alone with depression or anxiety represented a particularly vulnerable group. Unlike loneliness, which is difficult for primary care clinicians to identify, living alone is a readily discernible characteristic. Policy interventions addressing social isolation, such as community-based programs and mental health outreach, could mitigate the elevated suicide risk in this population.^16,57^

### Strengths and Limitations

A strength of this study is the use of the extensive NHIS database, which covers 97% of Korea’s population and provides robust statistical power and a wide array of detailed demographic, clinical, and socioeconomic information. The extended follow-up period of 11 years enhanced the reliability and validity of our findings. Furthermore, the inclusion of various demographic, lifestyle, and clinical factors in the analysis allows for a nuanced understanding of the associations among living arrangements, depression, anxiety, and suicide risk. Finally, living arrangements were assessed annually to minimize potential bias arising from misclassification of changes in household status.^42,47^

This study also had several limitations. The observational nature precludes establishing causality among living arrangements, psychiatric conditions, and suicide risk. While our study suggests that living arrangements and mental health were associated with suicide risk, the causal direction remains unclear (eg, whether mental health worsens due to living alone or vice versa^22,58^). Although analyses stratified by follow-up duration were conducted to assess the robustness of our findings, living arrangements were classified only at baseline, potentially leading to misclassification and hindering the tracking of dynamic changes during follow-up. Moreover, while living arrangements were assessed annually, assessing the period of living alone between assessments was not possible. The group living together included diverse arrangements, which might have different associations with suicide risk.^35^ Key psychosocial factors, such as previous suicide attempts, stressful life events, social support systems, quality of social interactions, and perceived loneliness or social isolation, were not included in the dataset.^59,60^ These limitations constrain the ability to comprehensively evaluate the broader psychosocial context and its association with suicide risk. Whether dynamic tracking of living arrangements and exploration of psychosocial factors, such as societal perceptions and coping strategies, could further clarify the association with these variables and suicide risk remains an area for future research. Reliance on *ICD-10* codes for psychiatric conditions might result in underreporting or misclassification due to the lack of systematic mental health assessments. The stigma surrounding mental disorders, particularly in Asian countries, could exacerbate barriers to seeking treatment and contribute to underdiagnosis, which may have underestimated the true prevalence in our cohort.^61,62,63^ Finally, this study relied on NHIS data representing the Korean population. However, Korea’s unique cultural, social, and health care characteristics may have influenced the observed associations, limiting the generalizability of our findings to other sociocultural and health care contexts.

## Conclusions

In this cohort study of 3 764 279 individuals, living alone combined with depression or anxiety was associated with an increased risk of suicide. These findings highlight the importance of considering living arrangements in individuals with depression or anxiety, especially for specific demographic groups, such as middle-aged individuals and men, in suicide risk assessments. Targeted interventions addressing these factors together are crucial to mitigate risk. Future research should explore these associations in diverse populations and investigate the potential benefits of tailored interventions.

## Notes

Supplement 1. **eTable 1.** Definitions and Descriptions of Variables Used in the Study **eTable 2.** Mortality, Suicide Rates, and Follow-Up Duration **eTable 3.** Interaction Analysis Between Living Arrangements and Mental Health Conditions (Depression or Anxiety) on Suicide Risk **eTable 4.** Hazard Ratios (HRs) and 95% Confidence Intervals (CIs) for Suicide Risk by Living Arrangements, Depression, and Anxiety, Stratified by Follow-Up Duration **eTable 5.** Hazard Ratios (HRs) and 95% Confidence Intervals (CIs) for Suicide Risk by Duration of Living Alone and Psychiatric Conditions: Sensitivity Analysis **eTable 6.** Comparison of Suicide Rates and Risk Among Individuals Excluded for Missing Data and Those Living Alone: Sensitivity Analysis

Supplement 2.
Data Sharing Statement
