# Supplementary material for: Suicide Risk and Living Alone With Depression or Anxiety
Source: JAMA Netw Open. 2025 Mar 26;8(3):e251227. doi: 10.1001/jamanetworkopen.2025.1227 (PMC11947838; doi:10.1001/jamanetworkopen.2025.1227)
Supplement: Supplement 1. — eTable 1. Definitions and Descriptions of Variables Used in the Study eTable 2. Mortality, Suicide Rates, and Follow-Up Duration eTable 3. Interaction Analysis Between Living Arrangements and Mental Health Conditions (Depression or Anxiety) on Suicide Risk eTable 4. Hazard Ratios (HRs) and 95% Confidence Intervals (CIs) for Suicide Risk by Living Arrangements, Depression, and Anxiety, Stratified by Follow-Up Duration eTable 5. Hazard Ratios (HRs) and 95% Confidence Intervals (CIs) for Suicide Risk by Duration of Living Alone and Psychiatric Conditions: Sensitivity Analysis eTable 6. Comparison of Suicide Rates and Risk Among Individuals Excluded for Missing Data and Those Living Alone: Sensitivity Analysis [file jamanetwopen-e251227-s001.pdf]

Supplemental Online Content

Moon DU, Kim H, Jung JH, Han K, Jeon HJ. Suicide risk and living alone with depression or anxiety. *JAMA Netw Open*. 2025;8(3):e251227. doi:10.1001/jamanetworkopen.2025.1227

eTable 1. Definitions and Descriptions of Variables Used in the Study
eTable 2. Mortality, Suicide Rates, and Follow-Up Duration
eTable 3. Interaction Analysis Between Living Arrangements and Mental Health Conditions (Depression or Anxiety) on Suicide Risk
eTable 4. Hazard Ratios (HRs) and 95% Confidence Intervals (CIs) for Suicide Risk by Living Arrangements, Depression, and Anxiety, Stratified by Follow-Up Duration
eTable 5. Hazard Ratios (HRs) and 95% Confidence Intervals (CIs) for Suicide Risk by Duration of Living Alone and Psychiatric Conditions: Sensitivity Analysis
eTable 6. Comparison of Suicide Rates and Risk Among Individuals Excluded for Missing Data and Those Living Alone: Sensitivity Analysis

This supplemental material has been provided by the authors to give readers additional information about their work.

© 2025 Moon DU et al. *JAMA Network Open*.

eTable 1. Definitions and Descriptions of Variables Used in the Study

Variables	Description
Low income	based on health insurance premiums (administration data), which are indicative of income levels in Korea: Individuals receiving medical aid or within the lowest quartile of income
Smoking status	Based on self-report. Categorized in never, current, and former smokers.
Alcohol consumption	Based on self-reported intake frequency. Non-drinkers indicated a response of "0" for drinking frequency on the question "How many days a week do you drink on average?"). We defined individuals who had consumed any amount of alcohol as alcohol drinkers.
Physical activity	Self-reported. Regular physical activity defined as engaging in regular moderate-intensity exercise for at least 30 minutes on 5 or more days per week or vigorous activity for at least 20 minutes on 3 or more days per week
Obesity	defined by a body mass index (BMI) of 25kg/m^2 or greater
Hypertension	claim for antihypertensive medication under the codes I10−I13 and I15 or recorded blood pressure readings (≥140/90 mmHg)
Diabetes	confirmed by at least one annual claim for antidiabetic medication under the codes E11−E14 or fasting glucose level (≥126 mg/dL).
Dyslipidemia	defined as having at least one yearly claim for lipid-lowering medication under code E78 or total cholesterol level (≥240 mg/dL).
Chronic Kidney Disease	classified based on ICD-10 codes N18, N19 or an estimated glomerular filtration rate below 60 mL/min/1.73 m^2, calculated according to the Modification of Diet in Renal Disease study equation[a]
Depression	identified by at least one claim for depressive disorders (ICD-10 codes F32, F33) within the year preceding the health examination date
Anxiety	identified by at least one claim for anxiety disorders (ICD-10 codes F40, F41) within the year preceding the health examination date
Post-traumatic stress disorder	identified by at least one claim for post-traumatic disorder (ICD-10 codes F43.1) within the year preceding the health examination date
Schizophrenia spectrum disoder	identified by at least one claim for schizophrenia spectrum disorder (ICD-10 codes F20-29) within the year preceding the health examination date
Substance use disorder	identified by at least one claim for substance use disorder (ICD-10 codes F10-19) within the year preceding the health examination date
Bipolar disorder	Identified by at least one claim with a primary diagnosis of bipolar disorders (ICD-10 codes F30, F31, F34.0) within the year preceding the health examination date.
Obsessive-compulsive disorder	identified by at least one claim for obsessive-compulsive disorder (ICD-10 codes F42) within the year preceding the health examination date
Personality disorder	identified by at least one claim for personality disorders (ICD-10 codes F60-61) within the year preceding the health examination date
Cancer	ICD-10 codes C00-C97
Cardiovascular disease	Included stroke and myocardial infarction. Myocardial infarction was defined as a record of ICD-10 codes I21 or I22 during hospitalization or these codes having been recorded at least twice. Stroke was defined as a record of ICD-10 codes I63 or I64 during hospitalization with claims for brain computed tomography or magnetic resonance imaging

[a]Levey AS, Coresh J, Greene T, et al. Expressing the Modification of Diet in Renal Disease Study equation for estimating glomerular filtration rate with standardized serum creatinine values. *Clin Chem*. 2007;53(4):766-772. doi:10.1373/clinchem.2006.077180

© 2025 Moon DU et al. *JAMA Network Open*.

eTable 2. Mortality, Suicide Rates, and Follow-Up Duration

A. Depression

	TOTAL	Living Together		Living Alone		*P*-value
		No Depression	Depression	No Depression	Depression	
Subjects (n)	3764279	3339384	104902	312435	7558	
Total Mortality (%)	235458 (6.26)	204441 (6.12)	15619 (14.89)	14442 (4.62)	956 (12.65)	<.001
Suicide (%)	11648 (0.31)	9536 (0.29)	955 (0.91)	1065 (0.34)	92 (1.22)	<.001
Follow Up Duration (person-years)						
Mean (SD)	11.05 (1.46)	11.06 (1.44)	10.66 (2.23)	11.12 (1.25)	10.77 (2.04)	<.001
Median [Q1-Q3]	11.29 [11.10-11.56]	11.29 [11.10-11.56]	11.33 [11.06-11.62]	11.28 [11.10-11.54]	11.3 [11.07-11.58]	<.001

B. Anxiety

	TOTAL	Living Together		Living Alone		*P*-value
		No Anxiety	Anxiety	No Anxiety	Anxiety	
Subjects (n)	3764279	3227854	216432	304120	15873	
Total Mortality (%)	235458 (6.26)	193474 (5.99)	26586 (12.28)	13704 (4.51)	1694 (10.67)	<.001
Suicide (%)	11648 (0.31)	9289 (0.29)	1202 (0.56)	1052 (0.35)	105 (0.66)	<.0001
Follow Up Duration (person-years)						
Mean (SD)	11.05 (1.46)	11.07 (1.43)	10.80 (2.02)	11.12 (1.24)	10.87 (1.86)	<.001
Median [Q1-Q3]	11.29 [11.10-11.56]	11.29 [11.10-11.56]	11.34 [11.08-11.62]	11.28 [11.1-11.54]	11.31 [11.08-11.58]	<.001

Table displays mortality and suicide rates (%) along with follow-up duration (person-years) categorized by psychiatric disorder (A. Depression, B. Anxiety) and living arrangement. Data show total counts, percentages, and follow-up metrics, with statistical significance noted by *P* Values.

© 2025 Moon DU et al. *JAMA Network Open*.

eTable 3. Interaction Analysis Between Living Arrangements and Mental Health Conditions (Depression or Anxiety) on Suicide Risk

[a]Incidence per 1000 person-years.

Living arrangements	Mental Disorder	Subjects (n)	Events (n)	Follow-up duration (person-years)	Incidence rate[a]	Hazard ratio (95% Confidence interval)				
						Model 1[b]	Model 2[c]	Model 3[d]	Model 4[e]	Model 5[f]
Living together	No Depression	3339384	9536	36938524	0.26	1(Ref.)	1(Ref.)	1(Ref.)	1(Ref.)	1(Ref.)
	Depression	104902	955	1117863	0.85	3.31 (3.10-3.53)	3.10 (2.90-3.32)	3.12 (2.92-3.34)	3.11 (2.91-3.33)	2.75 (2.56-2.95)
Living alone	No Depression	312435	1065	3473805	0.31	1(Ref.)	1(Ref.)	1(Ref.)	1(Ref.)	1(Ref.)
	Depression	7558	92	81381	1.13	3.68 (2.98-4.56)	3.28 (2.65-4.06)	3.23 (2.61-4.00)	3.22 (2.60-3.99)	2.85 (2.30-3.53)
P-value for interaction						0.342	0.621	0.756	0.758	0.756
Living together	No Anxiety	3227854	9289	35718619	0.26	1(Ref.)	1(Ref.)	1(Ref.)	1(Ref.)	1(Ref.)
	Anxiety	216432	1202	2337768	0.51	1.98 (1.86-2.10)	1.86 (1.75-1.98)	1.88 (1.77-2.00)	1.88 (1.77-2.00)	1.54 (1.45-1.64)
Living alone	No Anxiety	304120	1052	3382615	0.31	1(Ref.)	1(Ref.)	1(Ref.)	1(Ref.)	1(Ref.)
	Anxiety	15873	105	172579	0.61	1.96 (1.60-2.39)	1.75 (1.43-2.14)	1.74 (1.43-2.13)	1.74 (1.42-2.13)	1.43 (1.17-1.75)
P-value for interaction						0.932	0.566	0.472	0.477	0.488

[b]Model 1 was not adjusted.

[c]Model 2 was adjusted for sex and age.

[d]Model 3 was adjusted for sex, age, income, smoking status, alcohol consumption, physical activity, BMI, diabetes, hypertension, dyslipidemia, and chronic kidney disease.

[e]Model 4 was adjusted for sex, age, income, smoking status, alcohol consumption, physical activity, BMI, diabetes, hypertension, dyslipidemia, chronic kidney disease, and cancer.

© 2025 Moon DU et al. *JAMA Network Open*.

[f]Model 5 was adjusted for sex, age, income, smoking status, alcohol consumption, physical activity, BMI, diabetes, hypertension, dyslipidemia, chronic kidney disease, cancer, depression and anxiety.

© 2025 Moon DU et al. *JAMA Network Open*.

eTable 4. Hazard Ratios (HRs) and 95% Confidence Intervals (CIs) for Suicide Risk by Living Arrangements, Depression, and Anxiety, Stratified by Follow-Up Duration

Living Arrangement	Depression	Anxiety	Subjects (n)	Events (n)	Follow-up duration (person-years)	Incidence rate[a]	Hazard ratio (95% Confidence interval)			
							Model 1[b]	Model 2[c]	Model 3[d]	Model 4[e]
**Follow-up period: 1 year to <5 years**										
Living together	No	No	3159043	3138	12545982	0.25	1(Ref.)	1(Ref.)	1(Ref.)	1(Ref.)
		Yes	180341	354	711087	0.50	1.99 (1.78-2.22)	1.86 (1.67-2.08)	1.89 (1.69-2.11)	1.89 (1.69-2.11)
	Yes	No	68811	216	270050	0.80	3.20 (2.79-3.67)	2.94 (2.56-3.38)	2.96 (2.57-3.40)	2.95 (2.57-3.39)
		Yes	36091	179	141068	1.27	5.07 (4.36-5.90)	4.58 (3.93-5.34)	4.68 (4.02-5.46)	4.67 (4.00-5.44)
Living alone	No	No	299033	366	1189867	0.31	1.23 (1.10-1.37)	1.59 (1.42-1.77)	1.46 (1.31-1.63)	1.46 (1.31-1.63)
		Yes	13402	26	52975	0.49	1.96 (1.33-2.89)	2.27 (1.54-3.34)	2.08 (1.41-3.07)	2.08 (1.41-3.07)
	Yes	No	5087	22	20041	1.10	4.39 (2.89-6.68)	5.00 (3.28-7.60)	4.52 (2.97-6.88)	4.51 (2.96-6.87)
		Yes	2471	17	9677	1.76	7.03 (4.36-11.32)	7.67 (4.76-12.35)	6.97 (4.32-11.24)	6.95 (4.31-11.20)
**Follow-up period: 5 year to < 9 years**										
Living together	No	No	3110879	3041	12319014	0.25	1(Ref.)	1(Ref.)	1(Ref.)	1(Ref.)
		Yes	174889	262	685513	0.38	1.55 (1.37-1.76)	1.50 (1.32-1.71)	1.52 (1.34-1.73)	1.52 (1.34-1.73)
	Yes	No	66144	216	257852	0.84	3.39 (2.96-3.90)	3.26 (2.83-3.75)	3.28 (2.85-3.77)	3.27 (2.84-3.76)
		Yes	34358	122	133391	0.91	3.71 (3.09-4.44)	3.53 (2.94-4.24)	3.60 (2.99-4.32)	3.58 (2.98-4.30)
Living alone	No	No	295685	377	1173542	0.32	1.30 (1.17-1.45)	1.69 (1.51-1.88)	1.56 (1.40-1.74)	1.57 (1.40-1.75)

© 2025 Moon DU et al. *JAMA Network Open*.

	Yes	13066	20	51417	0.39	1.58 (1.02-2.45)	1.90 (1.22-2.95)	1.76 (1.13-2.74)	1.76 (1.13-2.74)
Yes	No	4934	15	19348	0.78	3.14 (1.89-5.22)	3.75 (2.26-6.23)	3.45 (2.08-5.73)	3.44 (2.07-5.71)
	Yes	2369	14	9234	1.52	6.14 (3.63-10.38)	7.06 (4.18-11.95)	6.48 (3.83-10.97)	6.46 (3.82-10.94)

[a]Incidence per 1000 person-years.

[b]Model 1 was not adjusted.

[c]Model 2 was adjusted for sex and age.

[d]Model 3 was adjusted for sex, age, income, smoking status, alcohol consumption, physical activity, BMI, diabetes, hypertension, dyslipidemia and chronic kidney disease.

[e]Model 4 was adjusted for sex, age, income, smoking status, alcohol consumption, physical activity, BMI, diabetes, hypertension, dyslipidemia, chronic kidney disease, and cancer.

© 2025 Moon DU et al. *JAMA Network Open*.

eTable 5. Hazard Ratios (HRs) and 95% Confidence Intervals (CIs) for Suicide Risk by Duration of Living Alone and Psychiatric Conditions: Sensitivity Analysis

Living Arrangement	Mental Disorder	Subjects (n)	Events (n)	Follow-up duration (person-years)	Incidence rate[a]	Hazard ratio (95% Confidence interval)				
						Model 1[b]	Model 2[c]	Model 3[d]	Model 4[e]	Model 5[f]
Living together	No Depression	2749839	8113	30341380	0.27	1 (Ref.)	1(Ref.)	1(Ref.)	1(Ref.)	1(Ref.)
	Depression	93414	856	992442	0.86	3.22 (3.00-3.46)	3.11 (2.89-3.34)	3.13 (2.91-3.36)	3.12 (2.90-3.35)	2.76 (2.56-2.97)
Living alone (1 year)	No Depression	218099	536	2438984	0.22	0.82 (0.75-0.90)	1.24 (1.13-1.35)	1.13(1.04-1.24)	1.13 (1.04-1.24)	1.13 (1.037,1.24)
	Depression	4560	44	49848	0.88	3.30 (2.45-4.44)	4.14 (3.08-5.57)	3.79 (2.82-5.10)	3.79 (2.81-5.09)	3.37 (2.52-4.54)
Living alone (2-4 years)	No Depression	371446	887	4158159	0.21	0.80 (0.75-0.86)	1.22 (1.14-1.31)	1.12 (1.05-1.21)	1.12 (1.05-1.21)	1.12 (1.05-1.21)
	Depression	6928	55	75574	0.73	2.72 (2.09-3.55)	3.46 (2.66-4.52)	3.20 (2.45-4.17)	3.19 (2.44-4.16)	2.85 (2.18-3.71)
Living alone (≥5 years)	No Depression	312435	1065	3473805	0.31	1.15 (1.08-1.22)	1.59 (1.49-1.69)	1.46 (1.36-1.55)	1.46 (1.36-1.55)	1.46 (1.36-1.56)
	Depression	7558	92	81390	1.13	4.23 (3.44-5.19)	5.16 (4.20-6.34)	4.69 (3.82-5.76)	4.68 (3.81-5.75)	4.14 (3.36-5.09)
Living together	No Anxiety	2651832	7888	29271566	0.27	1(Ref.)	1(Ref.)	1(Ref.)	1(Ref.)	1(Ref.)
	Anxiety	191421	1081	2062257	0.52	1.94 (1.82-2.07)	1.88 (1.76-2.01)	1.90 (1.78-2.03)	1.90 (1.78-2.02)	1.55 (1.45-1.66)
Living alone (1 year)	No Anxiety	213040	528	2382631	0.22	0.82 (0.75-0.90)	1.25 (1.14-1.36)	1.14 (1.05-1.25)	1.14 (1.04-1.25)	1.14 (1.04-1.25)
	Anxiety	9619	52	106201	0.49	1.82 (1.38-2.39)	2.35 (1.79-3.09)	2.17 (1.65-2.85)	2.17(1.65-2.85)	1.79 (1.36-2.36)
Living alone (2-4 years)	No Anxiety	362982	873	4064423	0.21	0.80 (0.74-0.86)	1.23 (1.14-1.32)	1.13 (1.05-1.22)	1.13 (1.05-1.22)	1.13 (1.05-1.21)
	Anxiety	15392	69	169310	0.41	1.51 (1.19-1.92)	1.99 (1.57-2.52)	1.84 (1.45-2.34)	1.84 (1.45-2.33)	1.54 (1.22-1.96)
Living alone (≥5 years)	No Anxiety	304120	1052	3382615	0.31	1.15 (1.08-1.23)	1.61 (1.51-1.72)	1.47 (1.38-1.57)	1.47 (1.38-1.57)	1.47 (1.38-1.57)

© 2025 Moon DU et al. *JAMA Network Open*.

	Anxiety	15873	105	172579	0.61	2.26 (1.87-2.74)	2.79 (2.30-3.38)	2.56 (2.11-3.10)	2.56 (2.11-3.10)	2.1 (1.73-2.55)

[a]Incidence per 1000 person-years.

[b]Model 1 was not adjusted.

[c]Model 2 was adjusted for sex and age.

[d]Model 3 was adjusted for sex, age, income, smoking status, alcohol consumption, physical activity, BMI, diabetes, hypertension, dyslipidemia and chronic kidney disease.

[e]Model 4 was adjusted for sex, age, income, smoking status, alcohol consumption, physical activity, BMI, diabetes, hypertension, dyslipidemia, chronic kidney disease, and cancer.

[f]Model 5 was adjusted for sex, age, income, smoking status, alcohol consumption, physical activity, BMI, diabetes, hypertension, dyslipidemia, chronic kidney disease, cancer, depression and anxiety.

© 2025 Moon DU et al. *JAMA Network Open*.

eTable 6. Comparison of Suicide Rates and Risk Among Individuals Excluded for Missing Data and Those Living Alone: Sensitivity Analysis

	Subjects (n)	Events (n)	Follow-up duration (person-years)	Incidence rate[a]	Hazard ratio (95% Confidence interval)				
					Model 1[b]	Model 2[c]	Model 3[d]	Model 4[e]	Model 5[f]
Excluded (Missing Data)	187119	642	2051674	0.31	1(Ref.)	1(Ref.)	1(Ref.)	1(Ref.)	1(Ref.)
Living alone	319993	1157	3555195	0.33	1.04 (0.95-1.15)	1.08 (0.98-1.18)	1.02 (0.92-1.12)	1.02 (0.93-1.13)	1.03 (0.94-1.14)

[a]Incidence per 1000 person-years.

[b]Model 1 was not adjusted.

[c]Model 2 was adjusted for sex and age.

[d]Model 3 was adjusted for sex, age, income, smoking status, alcohol consumption, physical activity, BMI, diabetes, hypertension, dyslipidemia and chronic kidney disease.

[e]Model 4 was adjusted for sex, age, income, smoking status, alcohol consumption, physical activity, BMI, diabetes, hypertension, dyslipidemia, chronic kidney disease, and cancer.

[f]Model 5 was adjusted for sex, age, income, smoking status, alcohol consumption, physical activity, BMI, diabetes, hypertension, dyslipidemia, chronic kidney disease, cancer, depression and anxiety.

© 2025 Moon DU et al. *JAMA Network Open*.
