# Supplementary material for: Suicide Risk and Living Alone With Depression or Anxiety
Source: JAMA Netw Open. 2025 Mar 26;8(3):e251227. doi: 10.1001/jamanetworkopen.2025.1227 (PMC11947838; doi:10.1001/jamanetworkopen.2025.1227)
Supplement: Supplement 2. — Data Sharing Statement [file jamanetwopen-e251227-s002.pdf]

Data Sharing Statement

Moon. Suicide Risk and Living Alone With Depression or Anxiety. *JAMA Netw Open*. Published March 20, 2025. doi:10.1001/jamanetworkopen.2025.1227

Data
Data available: No

Additional Information
Explanation for why data not available: Publicly available datasets were analyzed in this study. This data can be found here: https://nhiss.nhis.or.kr/.
